# Competitive Electrochemical Apta-Assay Based on cDNA–Ferrocene and MXenes for *Staphylococcus aureus* Surface Protein A Detection

**DOI:** 10.3390/bios14120636

**Published:** 2024-12-21

**Authors:** Ana-Maria Tătaru, Alexandra Canciu, Alin-Dan Chiorean, Ioana Runcan, Alexandru Radu, Mădălina Adriana Bordea, Maria Suciu, Mihaela Tertiș, Andreea Cernat, Cecilia Cristea

**Affiliations:** 1 Analytical Chemistry Department, Faculty of Pharmacy, Iuliu Haţieganu University of Medicine and Pharmacy, 4 Louis Pasteur St., 400349 Cluj-Napoca, Romania; ana.mari.tataru@elearn.umfcluj.ro (A.-M.T.); alexandra.canciu@elearn.umfcluj.ro (A.C.); runcan.ioana.ale@elearn.umfcluj.ro (I.R.); radu.alexandru.ioan@elearn.umfcluj.ro (A.R.); mihaela.tertis@umfcluj.ro (M.T.); 2 Department of Cell and Molecular Biology, Faculty of Medicine, Iuliu Hatieganu University of Medicine and Pharmacy, 400349 Cluj-Napoca, Romania; chiorean.alin@umfcluj.ro; 3 Emergency Clinical Hospital for Children, 400370 Cluj-Napoca, Romania; 4 Department of Microbiology, Faculty of Medicine, Iuliu Hatieganu University of Medicine and Pharmacy, 400349 Cluj-Napoca, Romania; madalina.bordea@umfcluj.ro; 5 Electron Microscopy Center “Prof. C. Craciun”, Faculty of Biology and Geology, “Babes-Bolyai” University, 5-7 Clinicilor St., 400006 Cluj-Napoca, Romania; maria.suciu@itim-cj.ro; 6 Electron Microscopy Integrated Laboratory, National Institute for Research and Development of Isotopic and Molecular Technologies, 67-103 Donath St., 400293 Cluj-Napoca, Romania

**Keywords:** aptasensor, *Staphylococcus aureus*, protein A, competitive assay, clinical samples, electrochemical detection

## Abstract

*Staphylococcus aureus* (*S. aureus*) represents one of the most frequent worldwide causes of morbidity and mortality due to an infectious agent. It is a part of the infamous ESKAPE group, which is highly connected with increased rates of healthcare-associated infections and antimicrobial resistance. *S. aureus* can cause a large variety of diseases. Protein A (PrA) is a cell-wall-anchored protein of *S. aureus* with multiple key roles in colonization and pathogenesis and can be considered as a marker of *S. aureus*. The development of aptasensors, having an aptamer as a specific biorecognition element, increases selectivity, especially when working with complex matrices. The association with state-of-the-art materials, such as MXenes, can further improve the analytical performance. A competitive aptasensor configuration based on a ferrocene (Fc)-labeled cDNA hybridized (cDNA-Fc S13) on a specific aptamer (APT) for PrA in the presence of MXene nanosheets was designed for the indirect detection of *S. aureus*. The aptasensor displayed a linear range of 10–125 nM, an LOD of 3.33 nM, and a response time under 40 min. This configuration has been tested in real samples from volunteers diagnosed with *S. aureus* infections with satisfactory results, enabling the perspective to develop decentralized devices for the rapid detection of bacterial strains.

## 1. Introduction

*Staphylococcus aureus (S. aureus)* represents one of the most frequent worldwide causes of morbidity and mortality due to an infectious agent. *S. aureus* is a well-known Gram-positive bacterium that can be both a commensal microorganism and a major human pathogen. The infections produced are particularly problematic because of frequently occurring antibiotic resistance in bacterial isolates, among which methicillin-resistant *S. aureus* (MRSA) is the most important. A consequence of MRSA infection is bacteremia, which, in 43% of the cases, can be complicated by exhibiting positive blood cultures, infective endocarditis, the presence of implanted protheses, follow-up blood cultures 48 h after the initial positive ones under a suitable antibiotic therapy, and metastatic sites of infection [1]. Recent studies have suggested that *S. aureus* is the main etiological agent of osteoarticular infections, including osteomyelitis, osteoarthritis, septic arthritis, and spondylodiscitis, in pediatric patients.

The past two decades have witnessed two shifts in the epidemiology of *S. aureus* infections: first, a growing number of healthcare-associated infections, particularly seen in infective endocarditis and prosthetic device infections, and second, an epidemic of community-associated skin and soft-tissue infections (including abscesses) driven by strains with certain virulence factors and resistance to *β*-lactam antibiotics. These infections occur mostly by hematogenous spread from bacteremia, with high risks of severe sepsis and suppurative complications, which can lead to sequalae, such as bone necrosis, cartilage damage, or growth impairment of long bones [1,2,3,4,5]. The rates of MRSA among clinical isolates vary greatly by country, ranging from single-digit rates in Scandinavian countries to over 50% in the U.S. and China. Although hospital-associated MRSA infections are on the decline in the U.S., Western Europe, Japan, and China, likely because of increased hygiene and surveillance measures, they are still on the rise in poorly developed countries, especially in Africa. In the U.S., the mortality caused by MRSA, recently reported by the Centers for Disease Control and Prevention (CDC), was estimated at ~20,000 cases in 2023 [6,7]. In all 16 countries submitting data to the European CDC in 2021, the majority of the identified isolates were attributed to *Escherichia coli* (37.9%), followed by *S. aureus* (17.2%) and *Klebsiella pneumoniae* (14.9%) [8]. Romania is one of the countries with the highest prevalence of MRSA in Europe, ranging from approximately 30% up to 70% [9]. According to the European CDC surveillance report “*Point prevalence survey of healthcare-associated infections and antimicrobial use in European acute care hospitals, 2022–2023*”, twenty-four EU/EEA countries reported at least ten *S. aureus* isolates with known antimicrobial susceptibility testing results for methicillin. Eleven countries reported less than 20% MRSA in *S. aureus* isolates from healthcare-associated infections, while in Romania, 73.2% of the *S. aureus* isolates were MRSA [10].

The United Nations 2030 Agenda for Sustainable Development includes seventeen goals adopted by all the member countries, as a global partnership, to reduce poverty by promoting strategies for health and education, reduce inequality, and enhance economic growth. The third goal of the agenda, “*Good health and well-being*”, strives to reduce mortality in under five years by tackling it with effective HIV treatments and childhood vaccinations and aims to achieve universal health coverage. Hence, the rapid detection of bacteria by alternative methods has the potential to reduce antimicrobial resistance and the prevalence of healthcare-associated infections and to have a positive impact on the healthcare system [11,12]. Electrochemical sensors have the capacity to provide highly specific and swift responses and can be easily integrated into rapid sensing devices, like point-of-use (POU)/point-of-care (POC) devices, suitable for biomedical applications [12].

Recently, MXenes have emerged as a novel class of 2D-layered nanomaterials that were reported for the first time in 2011 by Naguib et al. [13,14]. They are described by the general formula M_n+1_X_n_T_x_, in which M represents an early-transition metal, X stands for carbon or nitrogen, and T_x_ stands for surface functional groups. As a new material for sensor development, MXene nanosheets display performances superior to those of graphene, especially because of their high conductivity and chemical/mechanical stabilities. Both are 2D materials, but the presence in MXenes of other functional groups, such as –F, –OH, =O, and –Cl, contributes to their large active surface areas and hydrophilic nature, an advantage toward health applications. These functional groups have the capacity to interact with bioelements for specific targets via hydrogen bonds, van der Waals forces, and electrostatic and coordination bonds. Moreover, the fact that these materials can be synthesized from non-toxic compounds with easily tailorable properties is an advantage for applications in healthcare and food safety [15]. Despite their outstanding features, these materials present some challenges, such as being prone to oxidation due to light and temperature and having relatively short shelf lives in dispersions [16,17]. Despite the fact that graphene continues to be included in electrochemical sensor development [18,19,20,21,22,23], MXenes are foreseen to be employed in various configurations, especially those with a biomimetic element. Their high conductivity is very important in the detection of pathogens, especially via electrochemical aptasensors, where bioelements/proteins/blocking agents are included and decrease the electron transfer rate and generate low-intensity signals [24]. Moreover, the background noise, non-specific interactions, and the presence of other molecules could contribute to the same phenomena and diminish the analytical performances of the sensors. Ranging from health applications (like cancer diagnostics and monitoring [25,26,27], infectious diseases [28], diabetes [29], and cardiovascular diseases [30]) to food safety [31], agriculture, and environmental [16,32,33] applications, MXenes have proven their versatility. However, despite up-to-date advances in this field, MXene-based aptasensors are still lacking when referred to in healthcare applications [15].

Regardless of their analytical performances, MXenes can be included in miniaturized devices, along with aptamers and nanomaterials, for the development of portable (apta)sensors, an important feature in biomedical applications [34]. Aptamers display excellent features as bioreceptors, with properties superior to those of antibodies, which make them important biomimetic elements in the design of electrochemical sensors with high levels of affinity for targets [35,36].

An important strategy in aptasensor elaboration is represented by the design of competitive assays. In this case, a nanomaterial and a complementary DNA probe (also known as cDNA) are used to amplify the electrochemical signal. The labeled DNA probe is specifically bound to the aptamer (APT), which is often immobilized on the surface of the electrodes. Nanomaterials act as amplifiers of the signal by enhancing the active surface area and promoting the electron transfer rate. When the target is added to the configuration, the competition between the target and cDNA for the specific APT is triggered, and the complex cDNA-APT is easily dissociated. Hence, the target can specifically bind to the APT and generate a decrease in the analytical signal intensity after the removal of the labeled cDNA. Additionally, when nanomaterials such as graphene or MXenes are used, the current drop is often higher, and the analytical performances are further improved [34,37,38]. MXenes have good biocompatibility and are suitable for the assessment of biological samples.

Lately, several electrochemical aptasensor configurations have been developed for the rapid detection of pathogenic bacteria, mainly those involved in healthcare-acquired infections, such as *Escherichia coli* [39], *Acinetobacter baumannii* [40], *S. aureus* [41], and *Pseudomonas aeruginosa* [42], focusing either on the detection of whole bacteria or targets, such as Protein A (PrA), from *S. aureus*, but this approach is still being researched to be included in medical practice.

Building upon our previous work on detecting PrA as a marker of *S. aureus,* we have designed a competitive aptasensor configuration employing a ferrocene (Fc)-labeled cDNA (cDNA-Fc S13) hybridized on the specific APT for PrA. This step was achieved in the presence of MXene nanosheets, a state-of-the-art material, thereby ensuring a larger active surface area and promoting the electron transfer rate. Because of the different affinities between the APT and the cDNA and target, when PrA is incubated, the cDNA is practically removed, and the electrochemical signal intensity of the Fc label is substantially diminished. This aptasensor has been assessed using real samples from volunteers diagnosed with *S. aureus* infections.

## 2. Materials and Methods

The reagents were of analytical grade and used with no further purification or other pretreatments. Hydrochloric acid, ethanol, potassium chloride, sodium chloride, magnesium chloride, calcium chloride, potassium ferrocyanide (K_4_[Fe(CN)_6_]), potassium ferricyanide (K_3_[Fe(CN)_6_]), tris(hydroxymethyl)aminomethane (TRIS-HCl), tris(2-carboxyethyl)phosphine hydrochloride (TCEP), and 6-mercapto-1-hexanol (MCH) were acquired from Merck (Branchburg, NJ, USA). A titanium carbide (Ti_3_C_2_T_x_) (MXene) 1 mg/mL dispersion in propylene carbonate was synthesized by Nanochemazone (Leduc, AB, Canada). All the solutions were prepared in nuclease-free water (UltraPureTM Distilled Water DNase/RNase free) from Invitrogen–Thermo Fisher (Waltham, MA, USA). The APT and cDNA sequences with the corresponding modifications listed in Table 1 were synthetized by Eurogentec (Seraing, Belgium).

A 20 mM TRIS buffer (containing 20 mM TRIS-HCl, 100 mM NaCl, 100 mM KCl, 5 mM MgCl_2_⋅6H_2_O, and 1 mM CaCl_2_) was brought to pH 7.2 using a 0.1 M HCl solution and then filtered through a 0.45 μm Millipore^®^ filter membrane from Merck (Branchburg, NJ, USA) and used for the solution preparation and washing steps.

The platform was elaborated on AuSPEs with a gold counter electrode and a silver pseudoreference electrode from Metrohm–DropSens (Oviedo, Spain).

### 2.1. Aptasensor Elaboration Protocol

#### 2.1.1. APT Immobilization

The thiol-tethered APT (PA#2/8 (S1-58); 1 μM in 20 mM TRIS buffer; pH 7.2) selected from the literature [43] was immobilized on the Au working electrode’s surface using multipulse amperometry (MPA), where short alternative potential pulses of +0.5 V/Ag and −0.2 V/Ag, with a pulse duration of 30 ms, for an overall duration of 120 s, were applied following an optimized protocol developed by Canciu et al. [41]. After each modification step, the electrodes were rinsed 3 times with 50 μL of TRIS buffer to remove the unbound compounds.

#### 2.1.2. cDNA/MXene Deposition

A 1:1 mixture of cDNA (a 5 μM solution in TRIS) and the MXene (a 0.5 mg/mL suspension in propylene carbonate) was prepared. The cDNA-Fc-MXene mixture was deposited on the APT-modified working electrode’s surface and left for 30 min to allow the hybridization of the oligonucleotide sequence with its complementary strand. Subsequently, the electrodes were rinsed 3 times with 50 μL of TRIS buffer to remove the unbound cDNA fractions.

#### 2.1.3. Blocking Step

After the hybridization of cDNA-Fc S13 in the presence of the MXene, the unoccupied Au surface was blocked with 100 μM MCH in TRIS buffer (in 30% (*v*/*v*) absolute ethanol), with 30 μL of the blocking agent being incubated on the surface for 30 min at room temperature. The APT/cDNA-Fc-MXene/MCH electrodes were rinsed 3 times with 50 μL of TRIS buffer to remove the unbound thiol molecules.

### 2.2. Incubation of PrA

Different concentrations (10–500 nM) of PrA prepared in 20 mM TRIS (pH 7.2) were incubated for 30 min at room temperature on the APT/cDNA-Fc-MXene/MCH configuration. Following the same washing protocol, the electrodes were rinsed 3 times with 50 μL of TRIS buffer to remove the nonspecific bound protein and cDNA-Fc S13 released after the competitive interaction with the APT.

### 2.3. Real Samples

Real samples, consisting of hemocultures, were obtained from the clinical laboratory of the Emergency Clinical Hospital for Children (Cluj-Napoca, Romania), under ethics approval number 11839/12.09.2024, as follows:

Sample 1 consisted of an *S. aureus* standard strain (ATCC 25923). A number of bacterial colonies were suspended in 3 mL of isotonic saline solution (9 mg/mL NaCl) to a concentration equivalent to a 0.5 McFarland standard, using the VITEK DensiCHEK Display Base (bioMérieux, Marcy l’Étoile, France). Then, 1.5 mL of this suspension was injected into BACT/ALERT^®^ blood culture bottles and incubated in the BacT/ALERT 3D microbial identification system (bioMérieux, Durham, NC, USA) at 37 °C until a positive result was obtained;

Sample 2 was collected from an 8-year-old patient diagnosed with acute lymphoblastic leukemia and an infection caused by MSSA (methicillin-susceptible *S. aureus*);

Sample 3 was collected from a 12-year-old patient with a severe systemic infection caused by MRSA;

Sample 4 consisted of a strain of *Staphylococcus hominis* (*S. hominis*) isolated from a positive blood culture. Further analysis revealed that the positive blood culture was due to contamination, and no clinical infection with *S. hominis* was present.

Samples 2, 3, and 4 were collected from positive blood cultures. Each blood culture vial was incubated in the BacT/ALERT 3D at 37 °C until a positive result was signaled, after which the samples were analyzed using a VITEK 2 compact system and a Bruker MALDI Biotyper^®^ (Bruker Daltonics GmbH, Bremen, Germany), a system for bacterial identification.

All four samples were diluted to 1:100 with 20 mM TRIS (pH 7.2) prior incubation on the aptasensor, and the same protocol described in the case of PrA incubation was followed.

### 2.4. Electrochemical Experiments/Methods

The electrochemical experiments were performed using an Autolab PGSTAT302N potentiostat with NOVA 1.10 software (Metrohm Autolab, Utrecht, The Netherlands) and a SensitBT bipotentiostat with a PSTrace 5.9 software application (PalmSens B.V., Houten, The Netherlands) when real samples were concerned.

Each step of the fabrication process was assessed using cyclic voltammetry (CV) in 20 mM TRIS (pH 7.2) and electrochemical impedance spectroscopy (EIS) in 5 mM [Fe(CN)_6_]^3−/4−^ with a 0.1 M KCl redox probe. The CVs were carried out from −0.4 to 0.7 V/Ag at a scan rate of 0.05 V s^−1^, while the EIS experiments were performed in the frequency range between 0.1 MHz and 1 Hz, with a 0.01 V amplitude and at a DC potential determined using open circuit potentials. OriginPro 2022 (OriginPro v2023b; number GF3S5-6089-7180903; OriginLab Corporation, Northampton, MA, USA) was used for the data analysis, and BioRender.com Web application was used for the figure design.

### 2.5. SPR Measurements

The surface plasmon resonance (SPR) analysis was carried out using a BI-2500 SPR system (Biosensing Instrument Inc., Tempe, AZ, USA). For the affinity/interaction studies, gold sensor chips, with a 2 × 2 cm^2^ (400 mm^2^) area, purchased from Biosensing Instrument Inc. (Tempe, AZ, USA), were used. The experimental setup involved the immobilization of the APT sequence on the Au sensor chip through Au-S bond formation (by coating the sensor chip’s surface with 300 μL of 1 μM thiolated-APT solution and incubation overnight at 4 °C to allow for the formation of the self-assembled monolayer (SAM)). In the case of the competitive aptasensing strategy, the studied/investigated cDNA sequence (300 μL of 5 μM solution, the same concentration used in the elaboration of the electrochemical aptasensor platform) was incubated on the modified chip’s surface at room temperature for 1 h. After the hybridization, the Au-APT/cDNA surface was incubated with 300 μL of 100 μM MCH solution at room temperature for 30 min to ensure the blocking of the unoccupied active binding sites. After each modification step, the surface was rinsed with TRIS buffer to remove the unbound molecules. After the complete modification, the chip was mounted on the SPR prism.

The SPR experiments were carried out at 21 °C, using 10 mM TRIS (pH 7.2) as a running buffer at a constant flow rate of 60 μL/min. Two flow cells were used for the binding experiments, while a third flow cell was set as the reference channel. Initially, the running buffer was circulated through the channels to allow for sensor surface rehydration and for baseline equilibration. Prior to the target analyte (PrA) injection, the buffer solution was injected for blank signal subtraction. For the binding experiments, increasing concentrations of PrA (10, 50, 100, 200, 500, and 1000 nM) were sequentially injected for 300 s of exposure time followed by 60 s of dissociation. For processing the experimental data, BI Kinetics Analysis software v.3.10.6 (Biosensing Instrument Inc., Tempe, AZ, USA) and Scrubber 2.0 (BioLogic Software, Seyssinet-Pariset, France) were utilized.

### 2.6. SEM Experiments

For scanning electron microscopy (SEM) and energy-dispersive X-ray (EDX) analyses, the electrodes were mounted with carbon tape on sample stubs, and images were taken using a Hitachi 8230 SEM (Tokyo, Japan) at values between 10 and 30 kV, 10 µA, and a 13–15 mm working distance. Images were taken at three different points on the electrode’s surface at magnifications ranging from 30× to 50,000×. EDX analyses were conducted at the same places through an Oxford Instruments (Oxford, UK) EDS detector and were integrated using AZtec Software v.2.1 SP1/2018.

## 3. Results and Discussion

**Figure 1:**
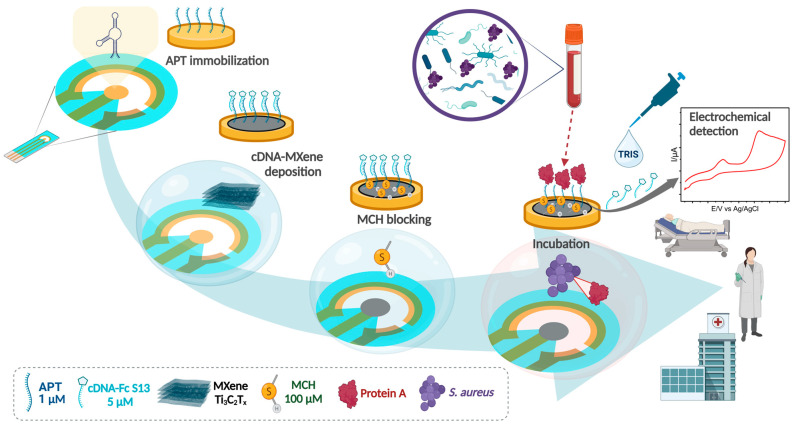
Aptasensor elaboration protocol and real-sample testing.

The first step in designing the competitive aptasensor involved the determination of the affinity between the APT and the target, as well as between APT/cDNA strands and the target by SPR. After the selection of the most suitable cDNA strand, the optimization was carried out for each step involved in the elaboration of the aptasensor as follows: the immobilization of the APT, the hybridization of the cDNA in the presence of the MXene nanosheets, and incubation with the PrA (Figure 1). The optimized configuration was selected for the analysis of clinical samples from patients with *S. aureus*-positive hemocultures.

### 3.1. SPR Studies

The intensity of the interaction between the APT and target molecule (PrA), also known as affinity, was determined from the thermodynamic and kinetic information obtained using the SPR technique [44]. The analysis of APT-PrA interactions was assessed based on the Langmuir isotherm binding model, assuming a 1:1 stoichiometry according to the equilibrium defined by Equation (1). The association (or on-rate) constant (*k_a_*) and the dissociation (or off-rate) constant (*k_d_*), along with the maximum binding response (R_max_) for each configuration were determined from the kinetic data analysis and are presented in Table 2. The binding affinity was evaluated as the equilibrium dissociation constant (*K_D_*), which was calculated as the ratio *k_d_*/*k_a_*, as defined in Equation (2).

APT + PrA ⇌ APT − PrA(1)

(2) $$\frac{k_{d}}{k_{a}}=\frac{\left[APT\right]\left[\mathrm{Pr}A\right]}{\left[APT-\mathrm{Pr}A\right]}=K_{D}$$

The determined *K_D_* values were in the nanomolar range (only for S19, it was close to the micromolar range), being comparable to the binding constants reported in the literature (for PrA without cDNA) [41]. The fastest association and the lowest *K_D_* value (17.51 ± 1.6 nM), meaning the highest affinity, were registered for the APT-cDNA S13 configuration.

**Figure 2:**
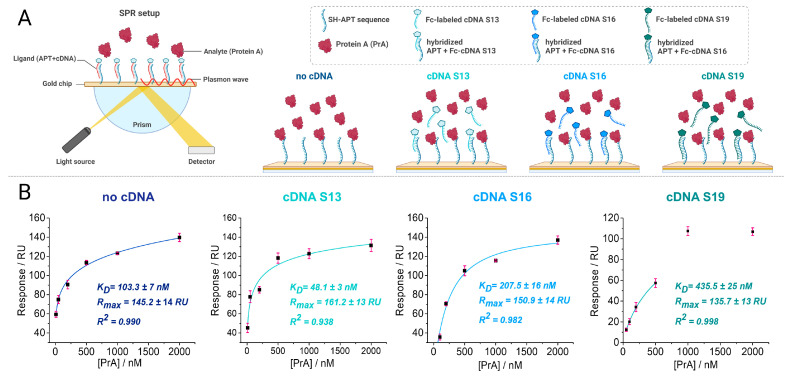
(**A**) Scheme of SPR detection principle using the different configurations as ligands for the target PrA. (**B**) Equilibrium data and corresponding fitting curves.

The steady-state binding data from the end of the association phase were plotted against the analyte concentration for each configuration, both the non-competitive and the competitive ones (Figure 2B). The data obtained from the binding assay under equilibrium conditions were fitted using a non-linear fitting model (Hill function) and with good correlation coefficients. The *K_D_* values were determined using the steady-state affinity model in the nanomolar range [41], with the configuration using the S13 sequence showcasing, again, the highest affinity (*K_D_* = 48.1 ± 3 nM). The highest dissociation rate was obtained for the APT-cDNA S19 and PrA complex, suggesting that the target is not bound as strongly as in the cases when the other sequences were used. The experimental data in the case of the S19 sequence could not be fitted for the same concentration range; the responses for the higher concentrations injected (1000 nM and 2000 nM) most likely were influenced by the non-specific adsorption of the target on the chip’s surface.

Adsorption on a chip’s surface causes changes in the SPR signal, which are expressed in resonance units (RUs).

Based on the binding interaction results, the length of the complementary strand seems to have an effect on the competitive interaction between the target protein and the immobilized APT. The *K_D_* values from both the kinetic and equilibrium analyses increased with the lengthening of the sequences: The lowest *K_D_* value was obtained for S13, then for the setup with only the APT and no cDNA, followed by S16 and S19. The cDNA sequence with the highest affinity (cDNA S13) was further considered for the competitive aptasensing design strategy.

### 3.2. Elaboration and Optimization Protocol

The competitive aptasensor has been designed based on our previous work [41], but unlike the initial configuration, an Fc-labeled cDNA strand was hybridized on the APT sequence to increase the specificity of the new aptasensor for the target. Also, to improve the analytical performance, MXene nanosheets were included in the immobilization protocol for cDNA-Fc S13. Each step was characterized using CV and EIS to display the modifications that took place after the immobilization of the APT, the hybridization of the cDNA-Fc S13 in the presence of the MXene, the blocking step, and, most importantly, the incubation of the target, PrA.

The APT deposition was performed using MPA with a duration of 120 s and a 30 ms pulse, following the optimized protocol previously reported by Canciu et al. [41]. Briefly, the SH-modified APT is immobilized on the surface of the AuSPE through the formation of Au-S bonds to obtain a self-assembled monolayer (SAM). Initially, a coordinative bond is formed between the SH group and Au, followed by a chemisorption process with proton dissociation and the generation of thiyl radicals. After the S-H bonds are dissociated, covalent Au-S bonds are formed. This reaction can occur spontaneously, or it can be promoted with the help of pulse-assisted techniques, such as MPA [45].

**Figure 3:**
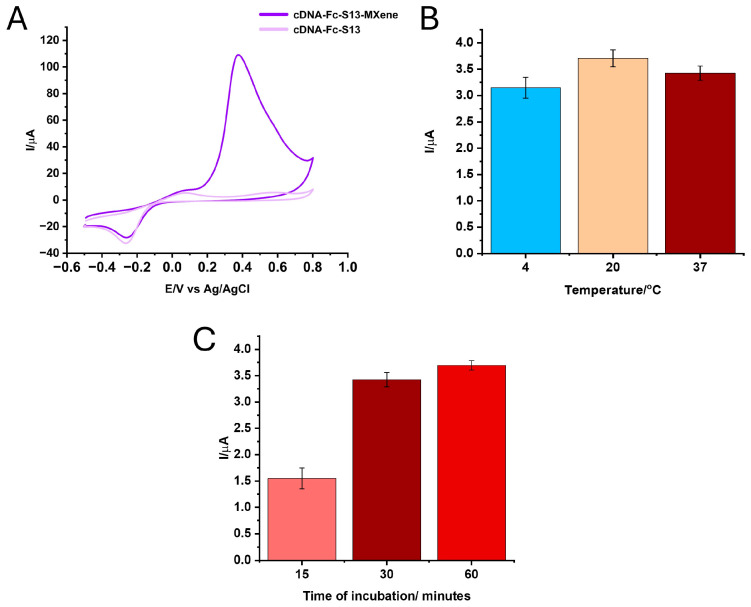
Optimization of the parameters for the hybridization of cDNA-Fc S13: (**A**) CV after the hybridization of cDNA-Fc S13 and in the presence of cDNA-Fc S13—MXene. CV signal (I (μA) ± RSD) of the anodic peak corresponding to the Fc label (**B**) for different temperatures of hybridization and (**C**) for different durations of the hybridization procedure.

The cDNA-Fc S13 was immobilized on the APT layer via hybridization at various temperatures (4, 20, and 37 °C) and timelapses (15, 30, and 60 min) in the presence of the MXene nanosheet suspension. In this case, the nanomaterial provides better conductivity, improving the analytical performance of the aptasensor. The optimization step was carried out by assessing the intensity of the anodic peak corresponding to the presence of Fc, as the label of the cDNA. The peak intensity registered at approximately 0.1 V/Ag in 20 mM TRIS buffer (pH 7.2) was higher when the hybridization procedure was performed at lower temperatures, like 4° and 20 °C, but overall, the experiments were more reproducible when hybridization at 37 °C was employed (Figure 3B). Concerning the duration of the hybridization, a 60 min timeframe gave the highest-intensity signal of the Fc with very good reproducibility (Figure 3C).

As mentioned, the cDNA-Fc S13 was immobilized in the presence of the titanium carbide MXene at a 1:1 dilution ratio. It can be clearly distinguished that the presence of the MXene determined a secondary anodic peak at around 0.4 V/Ag, confirming the immobilization of both the cDNA-Fc S13 and the nanomaterials on the Au electrode’s surface. No modification of the intensity of the peak corresponding to the Fc-labeled cDNA was observed after this step. However, the presence of the MXene was considered as necessary to increase the electron transfer rate, especially after the blocking step and incubation with the target, acting as an amplification element for the signal of the aptasensor [46].

#### 3.2.1. CV Experiments

CV experiments were performed in 20 mM TRIS (pH 7.2) to evaluate how each modification step influenced the electron transfer rate. In the case of the Au nonmodified electrode, two anodic peaks at 0.05 V and 0.53 V/Ag and a cathodic peak at −0.23 V/Ag, characteristic of the Au surface, were observed, as reported in other studies on gold-based surfaces [46]. After the APT deposition, no major modification of the current intensity was observed, with the exception of a slight anodic shift in the anodic peaks (at 0.10 V/Ag and 0.6 V/Ag). After this step, a cathodic peak was observed at −0.24 V/Ag, with a reduced intensity compared with that of the cathodic peak for the nonmodified electrode.

**Figure 4:**
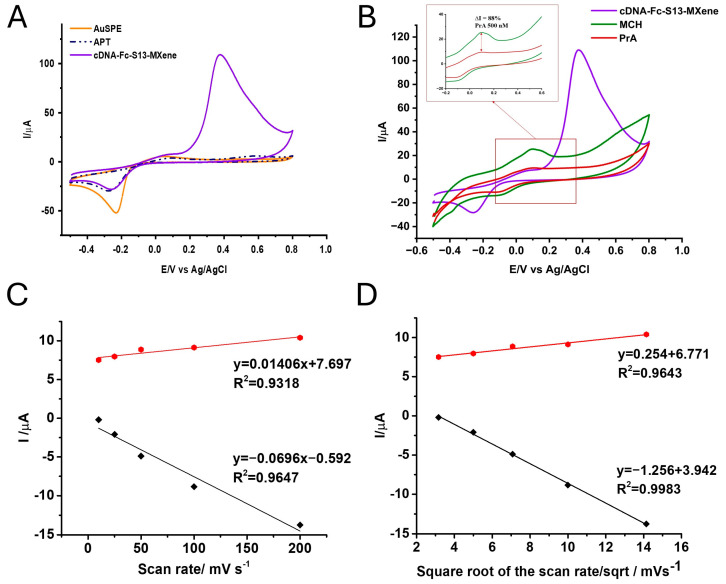
(**A**,**B**) CVs performed in 20 mM TRIS (pH 7.2) after each modification step in the elaboration of the aptasensor; (**C**) I vs. scan rate; (**D**) I vs. the square root of the scan rate, with error bars, for both anodic and cathodic peaks measured at different scan rates (10, 25, 50, 100, and 200 mV/s) from the CVs performed in 20 mM TRIS (pH 7.2) on the APT/cDNA-Fc S13-MXene after the blocking step with MCH.

After the deposition of the cDNA-MXene, the characteristic peak of the MXene was observed at around 0.38 V/Ag, with an intensity of approximately 100 µA. This peak was not observed when cDNA-Fc S13 was immobilized in the absence of the nanomaterial, and the presence of the 0.6 V/Ag anodic peak was attributed to the MXene, confirming the grafting of the cDNA and MXene (Figure 4A). As previously mentioned, the signal of the Fc was not observed after this step, possibly because of the presence of the APT on the electrode’s surface, which hindered the electron transfer or because of the high-capacitive current generated by the MXene nanosheets. However, after the blocking step with MCH, essential for preventing the non-specific adsorption of the analyte, the signal of the MXene nanosheets was diminished, probably because of overoxidation. In addition, the capacitive current was reduced, and a new anodic peak at 0.01 V/Ag and a cathodic one at −0.16 V/Ag were noticed and attributed to the oxidation/reduction of the Fc label from the cDNA S13 strand (Figure 4B).

The APT/cDNA-Fc-MXene configuration was incubated with standard solutions of PrA for 30 min at room temperature, and the signal corresponding to Fc decreased consecutively because of the specific interaction of PrA with the APT. More specifically, cDNA-Fc S13 is complementary to a partial sequence of the APT, and after the hybridization occurs, a portion of a double-stranded DNA sequence is generated. In the presence of PrA, the competition is triggered, the APT-cDNA double strand is dissociated, and a new complex structure, PrA-APT, with higher stability, is formed. This mechanism results in the removal of cDNA-Fc S13 from the surface of the electrodes and generates a drop in the current intensity corresponding to the Fc label, proportional to the amount of the PrA.

The interaction of the cDNA-MXene with the APT layer was also evaluated by studying the influence of the scan rate on the current intensity corresponding to the Fc label found on the cDNA. These experimental assays were performed only after the blocking step, when the signal of the Fc was amplified. Hence, the potential was scanned from −0.4 to 0.9 V/Ag at different scan rates as follows: 10, 25, 50, 75, and 100 mV/s.

As expected, the current intensity of the anodic signal increased proportionally with the scan rate (I_ox_ (µA) = 0.01406 × v + 7.697, R^2^ = 0.9318), confirming an adsorption-controlled process for Fc oxidation and proving indirectly the presence of the cDNA hybridized with the APT. The same trend was observed for the intensity of the reduction peak (I_red_ (µA) = −0.0696 × v − 0.5929, R^2^ = 0.9647) but at a much smaller scale, suggesting a low electron transfer rate, probably because of steric impediment or the blocking of the electrode’s surface (Figure 4C).

Additionally, when plotting the square root of the scan rate against the current intensity, a linear correlation, which confirms diffusion as the dominant process, was obtained (I_ox_ (µA) = 0.254 × v + 6.771, R^2^ = 0.9643 for the oxidation peaks and I_red_ (µA) = −1.256 × v + 3.942, R^2^ = 0.9983 for the reduction peaks) (Figure 4D).

Hence, we can affirm that both adsorption and diffusion phenomena are involved because there is no significant difference between the values of R^2^ (for the direct and square root plots) in both the oxidation and reduction processes. However, the blocking step is followed by the incubation of the PrA, and another rinsing step, which can physically remove the nonspecifically bound compounds, namely, cDNA-Fc S13 and the excess of the target.

#### 3.2.2. EIS Experiments

**Figure 5:**
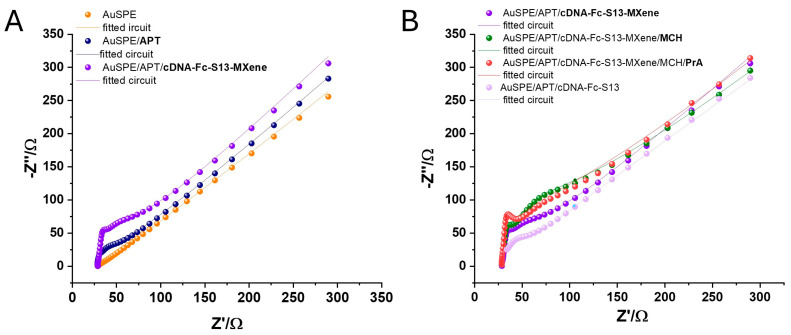
EIS performed in [Fe(CN)_6_]^3−/4−^ and 0.1 KCl after each modification step in the elaboration of the aptasensor: AuSPE surface (**A**) after APT immobilization and after cDNA-Fc-S13-MXene deposition; (**B**) after cDNA hybridization in the absence of the MXene, after cDNA-Fc-S13-MXene deposition, followed by MCH blocking and PrA incubation. The experimental data are represented by points, while the modeling data obtained from the fitting and simulation using the equivalent circuit are shown as lines.

The EIS experiments were performed in [Fe(CN)_6_]^3−/4−^ and 0.1 KCl and confirmed the CV data. Randles-type equivalent circuits were used to fit the model with the experimental data, using NOVA1.10.4 software. First, the equivalent circuit for the Au nonmodified surface was determined as [R_s_(C[R_ct_W])] (R_s_—resistance of the electrolyte solution, C—capacitance of the double layer, R_ct_—charge transfer resistance at the electrode/solution interface, and W—Warburg impedance). After the immobilization of the APT and subsequent to the formation of the thiol–Au bonds, the surface presented a different topography; hence, new circuit elements were included, and the circuit became [R_s_(Q[R_ct_W])(R_1_C_1_)(R_2_C_2_)]. The presence of a new constant phase element (Q) instead of C is attributed to the increased porosity of the surface, as observed in other studies [41]. The value of R_ct_ increased from 3.5 Ω to 78.3 Ω, confirming the immobilization of the APT on the Au surface, which reduced the electron transfer rate. The same circuit was fitted when cDNA-Fc S13 was hybridized on the APT surface, with no significant changes in the R_ct_ value. When cDNA-Fc S13-MXene was immobilized on the APT layer, the R_ct_ value increased to 125 Ω, and the fitted equivalent circuit was established as [R_s_(Q[R_ct_W])(R_1_C_1_)]. The presence of MXene nanosheets included in the topography probably reduced the porosity and the adsorption/desorption phenomena described by the (R_2_C_2_) elements (Figure 5A). As expected, after the MCH immobilization, the R_ct_ value increased because of the blocking effect of the molecule, which hindered the electron transport (from 125 Ω to 330 Ω), but no changes were recorded in the equivalent circuit. When 500 nM PrA was incubated on the APT/cDNA-Fc S13-MXene platform, a decrease in the R_ct_ to 188 Ω was observed, suggesting that in the presence the PrA, cDNA-Fc S13 was removed from the surface of the electrodes because of the superior affinity of the APT-PrA (Figure 5B). More specifically, following the incubation, a competition between PrA and cDNA-Fc S13 is triggered, the Fc-labeled cDNA is released, and the specific interaction between PrA and the APT sequence takes place. Hence, the modification of the R_ct_ value is determined by two distinct processes: the release of the cDNA and the capture of the target. The parameters of the Randles equivalent circuits are summarized in Table 3.

### 3.3. SEM Studies

**Figure 6:**
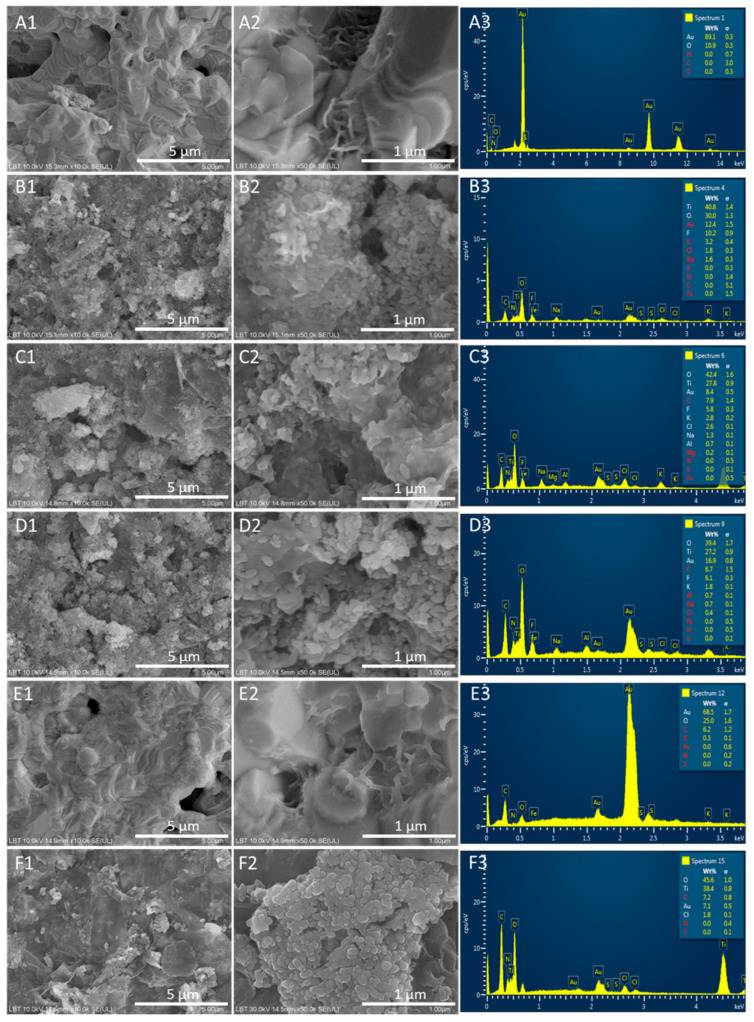
SEM and EDX results obtained for the electrode surfaces after each step of the aptasensor elaboration protocol. SEM images for the AuSPE functionalized with (**A1**,**A2**) APT; (**B1**,**B2**) APT/cDNA-MXene; (**C1**,**C2**) APT/cDNA-MXene/MCH; (**D1**,**D2**) APT/cDNA-MXene/MCH/PrA; (**E1**,**E2**) APT and cDNA; (**F1**,**F2**) MXene. Bars indicate 5 µm and 1 µm. (**A3**–**F3**) represent the corresponding EDX analyses.

From the SEM analysis of the gold electrode’s surface functionalized with APT (Figure 6A1,A2), a smooth surface with crystalline Au structures arranged in overlapping layers is observed. At higher magnifications, clumps of string-like structures are visible, likely representing the immobilized aptamers. The surface of the gold electrode, functionalized with APT and cDNA (Figure 6E1,E2), displayed a similar structural appearance, while the electrode functionalized solely with the MXene (Figure 6F1,F2) shows electron-transparent flakes, likely MXene nanosheets, alongside aggregations of the granulated material. The surfaces functionalized with APT/cDNA-MXene (Figure 6B1,B2), APT/cDNA-MXene/MCH (Figure 6C1,C2), and APT/cDNA-MXene/PrA (Figure 6D1,D2) exhibit a significantly grainy texture, with oval particles of 80–100 nm forming a nearly complete single-layer coverage of the 3D structure of the electrodes, which shows evident height variations.

In the EDX analysis, for the Au electrode functionalized with APT (Figure 6A3), Au constituted the majority (89 ± 0.3 wt.%), with O (10.9 ± 0.3 wt.%) being a minor component. The S and N peaks, corresponding to the chemical structure of the APT, were undetectable, likely lost in the background. For the electrode surface functionalized with APT and cDNA (Figure 6E3), a similar elemental distribution was found: Au (68.5 ± 1.7 wt.%), O (25 ± 1.6 wt.%), and C (6.2 ± 1.2 wt.%), corresponding to the APT and cDNA sequences. The iron peak, corresponding to the ferrocene group linked to the cDNA, was below the EDX detection threshold.

In the MXene-only-functionalized electrode (Figure 6F3), the Au base (7.1 ± 0.5 wt.%) was detected, along with elements from the MXene and propylene carbonate: Ti (38.4 ± 0.8 wt.%), O (45.6 ± 1.0 wt.%), C (7.2 ± 0.8 wt.%), and Cl (1.8 ± 0.1 wt.%). The surface functionalized with APT, cDNA, and the MXene (Figure 6B3) showed predominantly MXene elements: Ti (40.8 ± 1.4 wt.%), O (30 ± 1.2 wt.%), and Cl (1.8 ± 0.3 wt.%). Au (12.4 ± 1.5 wt.%) and elements from the buffer solution (K: 3.2 ± 0.4 wt.%, Na: 1.6 ± 0.3 wt.%, F: 10.2 ± 1.9 wt.%) were also quantified. After blocking with MCH (Figure 6C3) and PrA immobilization (Figure 6D3), additional elements, such as Al (0.7 ± 0.1 wt.%) and Mg (0.2 ± 0.1 wt.%), were detected.

### 3.4. Analytical Parameters

The signal intensity change (expressed as a percentage of the change in the signal intensity, ΔI (%), before and after the interaction with the PrA) is proportional to the quantity of the target that allowed for its indirect detection. As already described, after the specific interaction with the APT, cDNA-Fc was released, and the signal intensity of the Fc was greatly diminished.

**Figure 7:**
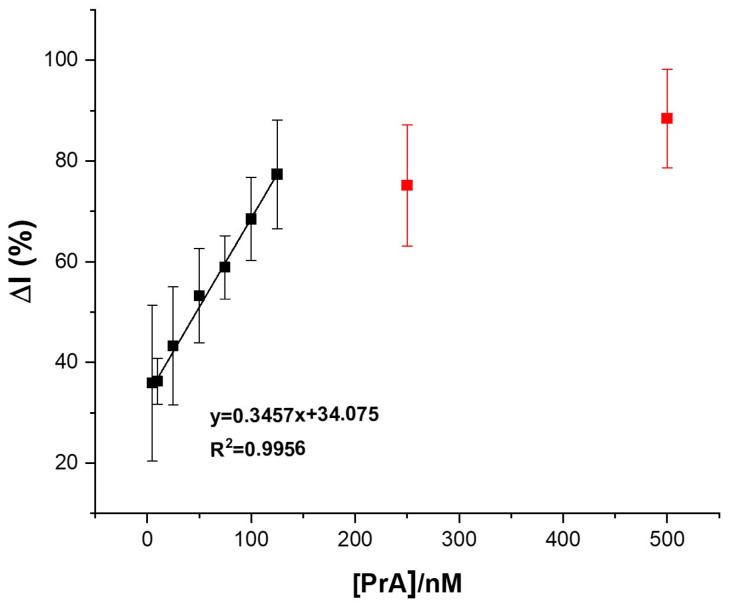
Variation in CV signal response (expressed as a percentage of the change in the signal intensity—ΔI (%) ± RSD) after incubation of the aptasensor with standard solutions of PrA in 20 mM TRIS buffer (pH 7.2) at different concentrations: 10–125 nM (black dots) and 250 and 500 nM (red dots).

A linear correlation was determined from 10 to 125 nM (I_ox_ (µA)= 0.3457 × [PrA] (nM) + 34.075, R^2^ = 0.9956) with a limit of detection (LOD) of 3.33 nM (S/N = 3) (Figure 7). When higher concentrations of PrA (250 and 500 nM) were incubated, a plateau shape of the curve was observed (Figure 7, red dots) and was associated with the saturation of the active sites available for the immobilization of the target.

### 3.5. Real-Sample Analysis

The performance of the aptasensor was assessed in complex matrices as follows: a standard *S. aureus* ATCC strain (Sample 1) and two real samples collected from patients diagnosed with *S. aureus* infection (Sample 2 and Sample 3 (MRSA)). A fourth real sample containing *S. hominis* (Sample 4) was also tested. *S. hominis* is known as the second most frequently isolated coagulase-negative staphylococci from healthy skin, and it may play a role in excluding pathogens, including *S. aureus*, from causing skin infections [47]. The morphology of this species is similar to that of *S. aureus*; however, its cell wall lacks PrA [48], and this specific strain was used as a control sample. The presence of bacteria in the samples was confirmed using plate culturing and MALDI-TOF MS.

**Figure 8:**
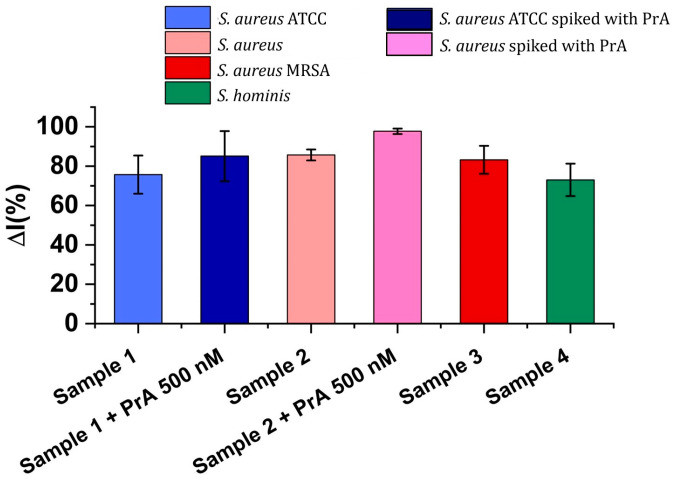
CV signal responses (ΔI (%) ± RSD) after 30 min of incubation of the aptasensor with real samples (diluted to 1/100 with TRIS buffer).

An important step in the electrochemical analysis of real samples is linked to the minimization of the matrix effect, a common issue when biological samples are concerned. Hence, a 1:100 dilution of the samples in 20 mM TRIS (pH 7.2) was performed prior to the analysis. The results are presented as percentages of the change in the signal intensity ΔI (%) before and after the interaction with the samples, as can be seen in Figure 8.

However, despite this pretreatment step, the aptasensors incubated with the real samples produced an intense signal, even in the absence of PrA, as can be seen for Sample 4, where no PrA was supposed to be present. Almost the same signal intensity was observed in the cases of the non-spiked samples (1, 2, and 3), which was attributed to the matrix effect. Furthermore, the recovery rates of the 500 nM PrA-spiked samples (Samples 1 and 2) were calculated at 11.5% (Sample 1) and 13.8% (Sample 2), using as reference the data from the calibration curve (for the 500 nM concentration). Although a very low recovery percentage was observed, this result was anticipated because of the matrix effect for the hemoculture samples, which has already been mentioned. However, aside from this issue, the aptasensor is promising for qualitative applications and can provide a rapid response when compared to those of conventional methods. Further studies will be conducted to reduce the interference of the matrix effect and to allow for the detection of lower PrA levels. Unfortunately, the literature data do not mention a quantitative correlation between PrA levels and CFUs, and this issue can be considered as a key point in this research domain and needs to be further investigated.

### 3.6. Stability and Reproducibility

**Figure 9:**
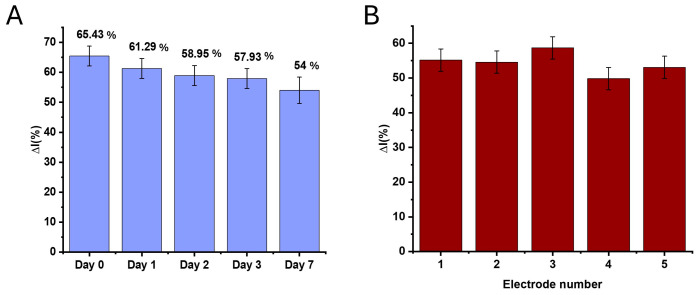
CV signal responses (ΔI (%) ± RSD) after the incubation of the aptasensor with 100 nM PrA for assessing (**A**) the stability over time (up to 7 days) and (**B**) the interelectrode reproducibility after the incubation with 50 nM PrA.

The stability was evaluated by storing the aptasensors at 4 °C (covered with TRIS buffer in a water-saturated atmosphere) and measuring the responses after 1, 2, 3, and 7 days in the presence of 100 nM PrA. The △I (%) value did not change significantly within the first 3 days, with the signal intensity being slightly reduced by approximately 12% (RSD = 2.3%), suggesting that the aptasensor had good stability in this timelapse (Figure 9A). However, after 7 days, the △I (%) value decreased by almost 17%, suggesting further degradation of the aptasensor.

The reproducibility was assessed with five different aptasensors in the presence of 50 nM PrA, and the RSD was calculated at 3.62%, revealing very good interelectrode reproducibility (Figure 9B).

## 4. Conclusions

A competitive aptasensor for the specific detection of PrA, a compound of the *S. aureus* cell wall, was designed based on a configuration that includes redox-marker-labeled cDNA and MXene nanosheets. The sensor was validated with real clinical samples from patients with *S. aureus* infections, highlighting its potential for accurate and efficient detection in complex biological matrices. Further studies need to be conducted to reduce the matrix effect, having in sight the perspective to be implemented in real practice. These results demonstrate that the developed aptasensor is a feasible tool for decentralized and rapid diagnostics, particularly in settings where timely detection of bacterial pathogens is critical for effective patient management.

## Figures and Tables

**Table 1:** List of oligonucleotide sequences used in the aptasensor elaboration.

Sequence Name	Nucleotide Sequence (from 5′ to 3′)	Modification
APT	ATA CCA GCT TAT TCA ATT AGC AAC ATG AGG GGG ATA GAG GGG GTG GGT TCT CTC GGC T	(CH_2_)_6_-SH-3′
cDNA S19	TAT GGT CGA ATA AGT TAA	5′-Fc
cDNA S16	TAT GGT CGA ATA AGT	5′-Fc
cDNA S13	TAT GGT CGA ATA	5′-Fc

Fc—ferrocene label.

**Table 2:** Binding parameters obtained from the kinetic analysis.

Configuration	*k_a_* (10^3^ M^−1^ s^−1^)	*k_d_* (10^−3^ s^−1^)	*R_max_* (RU)	*K_D_* (nM)
APT no cDNA	16.03 ± 0.27	1.44 ± 0.18	172.10 ± 24.7	90.32 ± 12.9
APT + cDNA S13	154.8 ± 53.1	2.54 ± 0.43	90.84 ± 5.9	17.51 ± 1.6
APT + cDNA S16	14.72 ± 2.9	2.69 ± 0.9	117.79 ± 18.7	177.52 ± 25.6
APT + cDNA S19	15.32 ± 7.6	1.19 ± 0.15	452.75 ± 8.2	952.84 ± 12.5

**Table 3:** The parameters of the Randles equivalent circuits.

	Circuit Elements	R_s_ (Ω)	R_ct_ (Ω)	C (nF)	Q_1_		W (mA/V)	R_1_ (Ω)	C_1_ (nF)	R_2_ (Ω)	C_2_ (nF)	χ^2^
Configuration					Y_o_ (μA/V)	N						
AuSPE		28.8	3.5	25.4	-	-	3.4	-	-	-	-	0.0086
AuSPE/APT		24.9	78.3	-	199	0.693	3.0	3.5	393	20.9	6.3	0.0054
AuSPE/APT/cDNA-Fc S13		29.7	69.1		1430	0.413	1.5	45.7	85.4	38.2	4.5	0.0104
AuSPE/APT/cDNA-Fc S13-MXene		22.5	125	-	111	0.881	2.8	82.2	7.9	-	-	0.0195
AuSPE/APT/cDNA-Fc S13-MXene/MCH		32.8	330	-	356	0.611	3.0	78.4	6.9	-	-	0.0108
AuSPE APT/cDNA-Fc S13-MXene/MCH/PrA 500 nM		32	188	-	9.4	0.873	2.6	101	284	-	-	0.0445

## Data Availability

Data are contained within the article.
